# *Rosa damascena* Mill. Essential Oil: Analysis of In Vitro and In Vivo Genotoxic and Cytotoxic Potentials by Employing Three Cytogenetic Endpoints

**DOI:** 10.3390/molecules30010078

**Published:** 2024-12-28

**Authors:** Tsvetelina Gerasimova, Svetla Gateva, Gabriele Jovtchev, Tsveta Angelova, Margarita Topashka-Ancheva, Ana Dobreva, Milka Mileva

**Affiliations:** 1 Institute of Biodiversity and Ecosystem Research, Bulgarian Academy of Sciences, 2 Gagarin Str., 1113 Sofia, Bulgaria; cvetij@yahoo.com (T.G.); spetkova2002@yahoo.co.uk (S.G.); angelova_ts@abv.bg (T.A.); topashka.mn@gmail.com (M.T.-A.); 2 Institute for Roses and Aromatic Plants, Agricultural Academy, 49 Osvobojdenie Blvd., 6100 Kazanlak, Bulgaria; anadobreva@abv.bg; 3 The Stephan Angeloff Institute of Microbiology, Bulgarian Academy of Sciences, 26 Acad. G. Bonchev Str., 1113 Sofia, Bulgaria; milkamileva@gmail.com

**Keywords:** *Rosa damascena* Mill., essential oil, genotoxicity, cytotoxicity, chromosomal aberrations, micronuclei, mitotic index, nuclear division index, in vitro test system, in vivo test system

## Abstract

The highly valued oil of *Rosa damascena* Mill. (Rosaceae), widely used in high perfumery, cosmetics, and other spheres of human life, obliges us to know and study the safety profile of the product obtained from the water–steam distillation of fresh rose petals. The genotoxicity of the essential oil (EsO) has not been thoroughly studied despite its wide range of applications. That predetermined the object of this study—to evaluate, through classical cytogenetic methods, the possible cytotoxic/genotoxic activities of *R. damascena* Mill. EsO (EsORdm) in three different test systems: plant root meristem cells, mammalian bone marrow cells, and human lymphocyte cultures. The rose essential oil showed varying concentration- and time-dependent cytotoxic and genotoxic effects depending on the test system used, and it was established that the oil showed moderate cytotoxicity in lymphocyte cultures and non-high cytotoxicity in ICR mice but none in barley. Both barley and human lymphocytes showed a genotoxic effect with a dose-dependent increase in chromosomal aberrations (CAs) and a substantial rise in micronucleus (MN) frequency, while no genotoxicity was observed in bone marrow cells at the applied concentrations. Human lymphocytes exhibited the highest susceptibility to cytotoxic and genotoxic actions of the EsO. As a valuable plant-derived aromatic product with versatile uses in human life, *R. damascena* Mill. essential oil should be used in an appropriate concentration range tailored to cellular sensitivity.

## 1. Introduction

The use of essential oils (EsOs) from various medicinal and ornamental plants in traditional and folk medicines has increased interest in research on their biological activities. A high-value EsO obtained utilizing the steam distillation of flowers of the Damask rose (*Rosa damascena* Mill. *f. trigintipetala* Dieck (Rosaceae)) is one of aromatic plants’ most expensive EsOs. The Damask rose is traditionally grown in the Valley of Roses in Central Bulgaria. Bulgarian rose production primarily focuses on *Rosa damascena* Mill. from Kazanlak, which ranks as one of the largest producers of rose essential oil worldwide. Rose essential oil is an extremely precious raw material in the perfume industry. Its other properties and benefits are widely recognized, making it a sought-after product and a valuable culture to cultivate. Rose essential oil and its products have well-known antibacterial and disinfectant properties [1,2]. The oils are reported to possess anticancer [3,4], anti-aging [5], antibacterial [6,7], and antimicrobial [1,8] activities. Essential oils are increasingly used as antibiotic-resistance-modifying agents [9]. Scientific data show that resistance reversal in bacteria underlies the synergistic effect of essential oils with conventional antibiotics [10]. The EsO from Bulgarian *R. damascena* Mill. can also be used as a potential therapeutic for herpes infections [11].

In fresh flowers, the essential oil yield [12] of the oil-bearing rose (*Rosa damascena* Mill.) is relatively low, around 0.3–0.4 mL kg^−1^, and can vary. Some studies exist about the chromatographic profiles of *R. damascena* Mill. from various regions of Bulgaria [13]. The chromatographic profile of the Bulgarian *R. damascena* EsO, derived from water–steam distillation from roses growing in the Kazanlak region (Valley of Roses) and used by us, was published by our co-author Dobreva et al. [14], with the international standard ISO 9842:2003 as a reference point [15]. The volatile compounds comprised mainly oxygenated monoterpenes (OMs), which are noteworthy among the identified compounds (56.5%), such as citronellol, geraniol, nerol, and linalool, followed by aliphatic hydrocarbons (AHs) (26.2%) with high molecular weights (C15–C27), such as nonadecane, heneicosane, 3-nonadecynetricosane, and eicosane, and minor concentrations of phenol derivatives (PDs) (2.9%), such as eugenol and methyleugenol.

The quality and properties of rose essential oil are influenced by the environmental conditions in which the plant grows [13,16]. Distillation methods, such as factory- and village-style distillations [17], are also essential. The balance of ingredients and chemical composition of EsOs play crucial roles in determining their effects [18]. The available literature information pertains predominantly to specific chemical compounds’ biological effects. In general, the cytotoxic activities of EsOs described by some authors are primarily because of the presence of phenols, aldehydes, and alcohols [19]. Polyphenol ingredients of plants, such as flavonoids, have potent antioxidant properties [20,21,22], but essential oils contain only some lipophilic molecules of this class. Wei and Shibamoto [20] found that rose essential oil, containing a high percentage of citronellol (34.2%), had a potent antioxidant capacity, over 50%, even at a low concentration of 500 μg/mL, according to the DPPH method. Other authors have reported that the composition of phenolic compounds in *R. damascena* extracts varies by sample origin [23]. Phenolic compounds appear to be at the core of the underlying biological properties of *R. damascena* extract [24], which can cause cellular death either by interacting with energy-generating enzymes or by causing protein denaturation [25].

The components geraniol and eugenol, found in rose EsO, have shown the potential to reduce the harmful effects of N-methyl-N’-nitro-N-nitrosoguanidine (MNNG) on barley meristem cells and human lymphocyte cultures [26]. Geraniol exhibits cytotoxic and antitumor effects against various cancer types [18,27]. Similarly, eugenol and its analogs have demonstrated inhibitory activity on cell growth in cancer cell lines [28]. Additionally, eugenol has shown promising results in reducing the incidence of MNNG-induced gastric tumors [29], making it a potential candidate for cancer remedy or prevention.

Essential oils can have cytotoxic effects on living cells, depending on their type and concentration [18,30], but they are generally non-genotoxic [3,31]. In certain instances, the abilities of essential oils to induce changes in the intracellular redox potential and mitochondrial dysfunction may be related to their capacities to exert antigenotoxic effects [18] and demonstrate an overt defense capacity [26,32]. The essential oil extracted from *Rosa damascena* Mill. *f. trigintipetala* Dieck has been found to exhibit antimutagenic activity against mitomycin C in normal human blood lymphocytes [3].

Investigating the safety of rose oil products commonly used in human practice is essential because of their popularity, leading to increasing scientific interest. However, much of the research into rose EsOs is still in its early stages, and an in-depth approach to the study of their biological activities has only been initiated in recent decades. This is particularly true regarding the cytotoxic effects on eukaryotic cells and underscores the need to evaluate their cytotoxicities thoroughly, which is crucial for their safe and effective use.

The cytotoxicity of the oil from *R. damascena* Mill. has been comparatively widely studied in various genotoxic assays [4,33,34]. However, the genotoxic properties of EsORdm have not been thoroughly studied, despite its wide range of applications and undeniable benefits. This lack of spatial research has motivated our study, which aims to fill the gaps in our understanding of the cytotoxic and genotoxic potentials of EsORdm. The assessment was carried out using classical cytogenetic methods in both in vitro and in vivo test systems, which included the chromosomal aberration assay and micronuclear assays. Using various test systems and appropriate endpoints would contribute to obtaining a more expressive and representative assessment of the rose essential oil’s cyto- and genotoxic effects.

## 2. Results

### 2.1. Cytotoxic Effect of R. damascena Mill. EsO

#### 2.1.1. Mitotic Index (MI)

**Figure 1:**
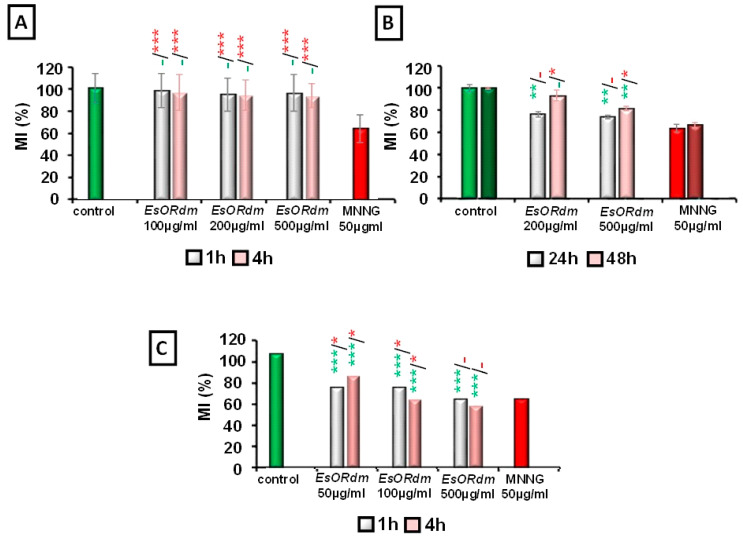
Cytotoxic activities of EsORdm assessed based on the values of MI in *H. vulgare* (**A**); mouse bone marrow cells (**B**); human lymphocytes (**C**). Mitotic activity (MI) was evaluated as a percentage of the negative control; * *p* < 0.05, ** *p* < 0.01, *** *p* < 0.001, and (–) non-significant versus the negative control (before the slash) versus the positive control, MNNG (after the slash).

Concerning the influence of the *R. damascena* Mill. EsO on the proliferative activity (Figure 1), no cytotoxic effect was observed in *H. vulgare* root tip meristems at concentrations of 100, 200, and 500 μg/mL for 1 and 4 h, compared to the untreated control (*p* > 0.05). Unlike barley, human lymphocytes were more sensitive (*p* < 0.001), with expressed cytotoxicity compared to the control values. The MI values slightly decreased with increasing EsO concentration (50, 100, and 500 μg/mL), which was more pronounced in variants with 4 h of treatment (at 500 mg/mL of EsO, the mitotic activity is 67% compared to that of the control). The lymphocyte cells showed concentration-dependent cytotoxicity—at 50 and 100 μg/mL (*p* < 0.05), as assessed using MI as an endpoint.

The MI values in the bone marrow cell population ranged from 74.11% to 93.13% mitotic activities compared to those of the untreated control animals—100%. The duration of the exposure after the EsO treatment, i.e., 24 and 48 h, as well as the doses tested (200 and 500 μg/mL), did not show significant suppression of mitotic activity (*p* > 0.05). The MI values were not concentration dependent (*p* > 0.05) between groups and slightly increased with increasing duration of the treatment. Almost all the EsO-treated groups had MI frequencies significantly different from those calculated for the untreated control (*p* < 0.01), except for the values calculated 48 h post 200 μg/mL of EsO supplementation (*p* > 0.05). No cytotoxicity was detected at the 48th hour of the 200 µg/mL of EsORdm treatment.

The data support the comparatively low cytotoxicity after treatment with EsORdm compared with that of the positive control group (MNNG 50 μg/mL) in *H. vulgare* cells. MNNG treatment induced a significant cytotoxic effect in the barley test system (*p* < 0.001). In bone marrow cells, the number of mitoses was highly reduced 24 h after EsO supplementation and was comparable with the MNNG data (*p* > 0.05). By contrast, the values in the 48 h groups differed slightly but significantly from the MNNG values (*p* < 0.05). The data obtained for lymphocyte cultures showed that the highest EsORdm concentration (500 μg/mL) was statistically indistinguishable from the MNNG data and, therefore, was cytotoxic.

The results from the in vivo and in vitro experiments indicate that EsORdm showed moderate cytotoxicities toward lymphocyte and bone marrow cells compared to MNNG (see Figure 1).

#### 2.1.2. PCE/(PCE + NCE) (%)

**Figure 2:**
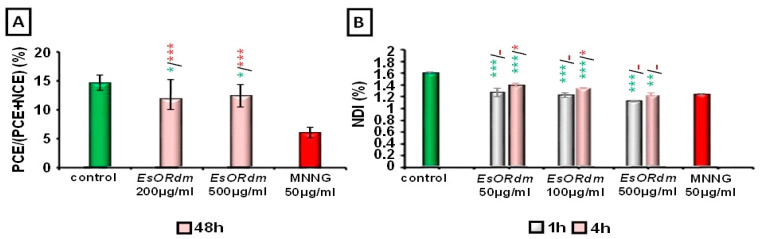
Cytotoxic activities of EsORdm assessed based on PCE/(PCE + NCE) ratio in ICR mice (**A**) and value of NDI in human lymphocytes (**B**); * *p* < 0.05, ** *p* < 0.01, *** *p* < 0.001, and (-) non-significant versus the negative control (before the slash) versus the positive control, MNNG (after the slash).

The ratio of the PCE to the total number of erythrocytes (PCE + NCE) was determined for each animal and used as another index of cytotoxicity (Figure 2A). These ratios (11.95 ± 3.0%/200 µg/mL and 12.28 ± 1.89%/500 µg/mL of EsORdm) do not yield a dose-dependent pattern in both oil-treated groups (*p* > 0.05). A slight but significant proliferation reduction was detected by comparison with the control group values (14.44 ± 1.27%) (*p* < 0.05). This slight cytotoxic effect was verified by the MNNG data (6.09 ± 0.93%) (*p* < 0.001).

#### 2.1.3. Nuclear Division Index (NDI)

The NDI was significantly reduced (*p* < 0.001) in the one-hour treatment scheme at all EsORdm concentrations tested (50 µg/mL 1.26% ± 0.07; 100 µg/mL 1.22% ± 0.04; 500 µg/mL 1.12% ± 0.10) vs. control values (1.59% ± 0.02) in human lymphocyte cells. The same pattern was observed for the 4-hour treatment (*p* < 0.01) (Figure 2B).

The NDIs were similar to those of the MNNG samples after 1 h of treatment (*p* > 0.05), but after 4 h, a significant increase in the index was found (*p* < 0.01) after the 50 and 100 µg/mL of EsORdm treatments. The NDI of the highest concentration (500 µg/mL) was identical to that of the MNNG value (1.23% ± 0.03), indicating a suppression of the activity of cellular division.

The data from the NDIs showed that EsORdm suppressed the activity of cellular division, and the results are like the MI data in human lymphocytes in vitro.

### 2.2. Genotoxic Effect of R. damascena Mill. EsO

#### 2.2.1. Induction of Chromosomal Aberrations (CAs)

**Figure 3:**
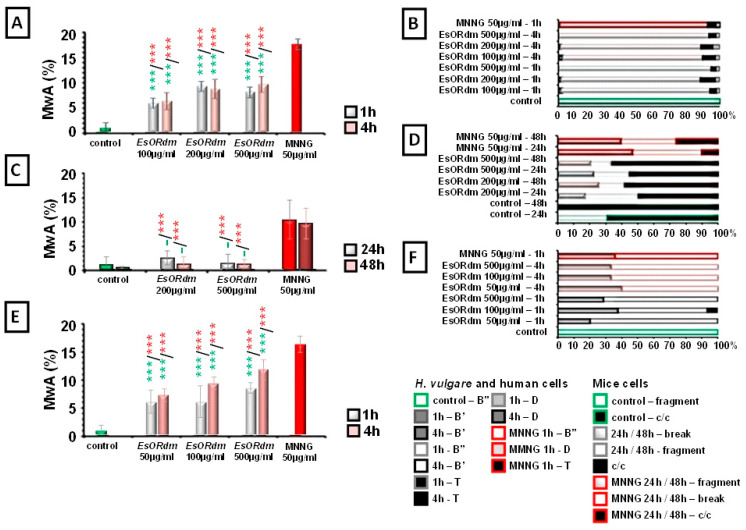
Genotoxicity results of EsORdm assessed based on the frequency of metaphases with aberrations (MwA) in *H. vulgare* (**A**), mouse bone marrow cells (**C**), and human lymphocytes (**E**). Distributions of aberrations observed post rose essential oil treatment in *H. vulgare* (**B**), mouse bone marrow cells (**D**), and human lymphocytes (**F**). The statistical significance is denoted by *** *p* < 0.001, and (–) non-significant versus negative control (before the slash) versus positive control, MNNG (after the slash).

All three test systems underwent testing to assess the induction of CAs after being treated with varying concentrations of EsORdm. The results are presented in Figure 3.

In the plant test system (Figure 3A) and human lymphocytes (Figure 3E), EsORdm showed a significant increase in the total number of metaphases with aberrations as compared to the negative control (*p* < 0.001) at all the concentrations tested. EsORdm induced CAs in plant meristem cells, with frequencies ranging from 6.07% ± 1.78 for 100 µg/mL to 9.47% ± 1.98 for 200 µg/mL and 8.27% ± 2.50 for 500 µg/mL after 1 h of treatment. After 4 h of treatment, the frequencies observed were similar across all three concentrations tested, ranging from 6.47% ± 1.63 to 8.93% ± 1.87 and 9.93% ± 1.53, respectively. In lymphocyte cells, concentrations of 50, 100, and 500 µg/mL (1 h) induced chromosomal damages in ranges from 6.00% ± 2.0 to 6.00% ± 2.80 and 8.40% ± 0.90, respectively, without any concentration dependence. Concentrations of 50 and 100 µg/mL/4 h induced a range from 7.20% ± 1.10 to 9.20% ± 1.10. The highest value of the aberrations reported in human lymphocytes was in the sample affected by 500 µg/mL of EsORdm (11.60% ± 1.70) after 4 h of treatment.

In contrast to these results, in the bone marrow cells, no clastogenic effect (*p* > 0.05) was observed; in fact, all the mean values of the aberrant cells (between 1.25 and 2.5%) were quite close to those of the untreated control group’s data (1.14% ± 1.57) (see Figure 3C). There was no dose-dependent statistically significant difference between the two EsO concentrations tested. The number of aberrations did not increase significantly with the treatment period extension (from 24 to 48 h) (*p* > 0.05).

In this experimental scheme, the EsORdm genotoxic effect was more prominent in human lymphocyte cultures than in *H. vulgare* and ICR mice.

The frequencies of CAs in all three test systems were far lower (*p* < 0.001) than those received after MNNG treatment (18.07% ± 3.5 in *H. vulgare*, 10.28% ± 3.92 in bone marrow cells, and 16.00% ± 1.40 in human lymphocytes) at all the EsORdm concentrations tested.

An analysis of the distributions of CAs showed that in barley, EsORdm induced mainly isochromatid breaks (B″) (Figure 3B), with percentages from 86.34% to 93.34% and significantly lower rates of translocations (Ts), ranging from 3.01% to 9.32%. Cytogenetic analysis in bone marrow cells revealed minor frequencies of breaks, fragments, translocations, and other chromosomal rearrangements in the bone marrow metaphases (Figure 3D). Centromeres/centromeric fusions were the most common type of chromosomal aberration (from 50 to 66% of all the rearrangements). The chromatid types of aberrations (breaks and fragments) in metaphases were almost equally presented across all the experimental groups (from 33 to 50%). In human lymphocytes (Figure 3F), the observed CAs were predominantly isochromatid breaks (B″) in the range from 56.2 to 80%, followed by chromatid breaks (B′) from 20 to 40%.

**Figure 4:**
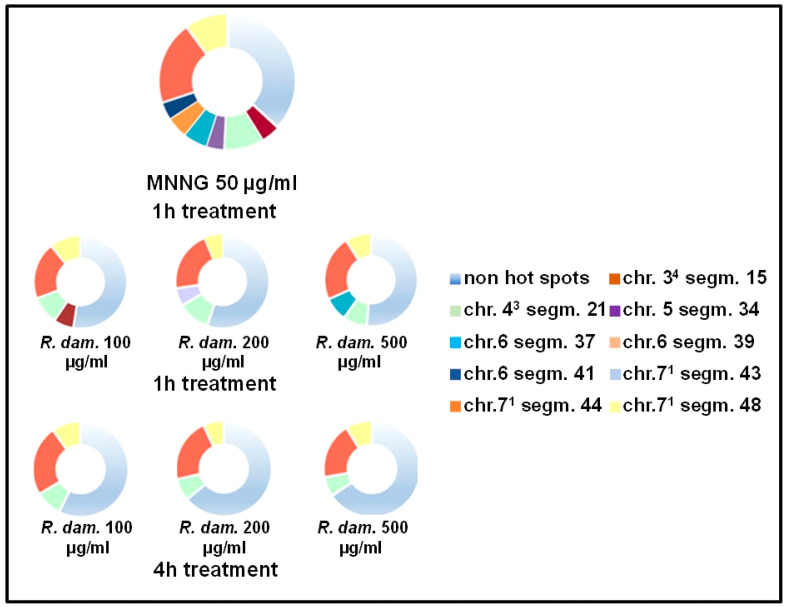
Aberration “hot spots” observed in the reconstructed * karyotype (MK14/2034) of *H. vulgare* after treatment with three different concentrations of EsORdm. * The karyotype of *H. vulgare* was reconstructed by combining two simple reciprocal translocations: one between parts of chromosomes 1 and 7 and the other between parts of chromosomes 3 and 4.

Identifying aberration “hot spots” in the reconstructed karyotype MK14/2034 can provide additional information on the EsO’s effect on *H. vulgare* root meristem cells. In the conducted experiments for the single treatment with MNNG (50 μg/mL), eight hot spots were observed out of the 48 inspected segments (Figure 4). It was found that the yields of aberration “hot spots” induced by different EsORdm concentrations were considerably lower than the number of hot spots induced by MNNG (namely, four “hot spots” after a one-hour treatment with EsO and three “hot spots” after 4 h of treatment, respectively). Segment 21 of chromosome 4^3^ (between 6.70 and 11.20%) was affected by all the tested concentrations for both treatment intervals (1 and 4 h), as well as segments 44 and 48 of chromosome 7^1^ (from 18.90 to 23.80% and from 6.20 to 10.90%, respectively). In the one-hour treatment variants, an additional “aberration hot spot” was detected in segment 15 of chromosome 3^4^ (6.90%, 100 µg/mL), as well as in segment 37 of chromosome 6 (8.30%, 500 µg/mL) and segment 43 of chromosome 7^1^ (6.20%, 200 µg/mL).

**Figure 5:**
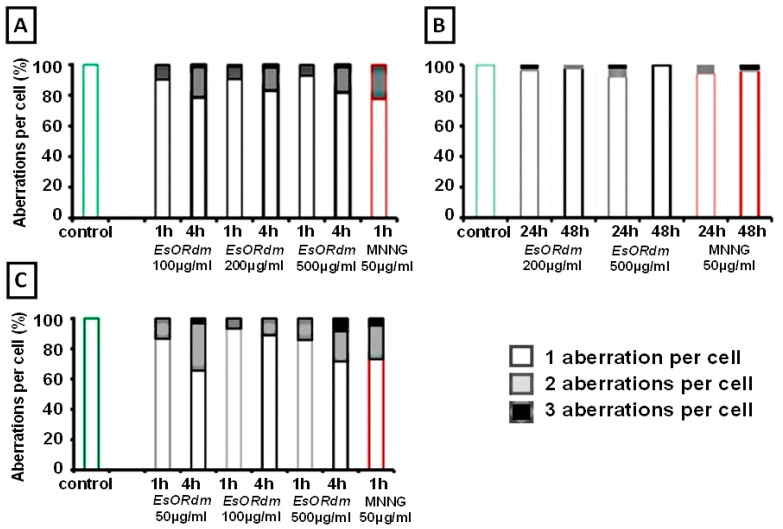
Aberrations per cell, as observed in *H. vulgare* (**A**) and human lymphocytes (**C**) one and four h after EsORdm supplementation and in ICR mice (**B**) at the 24th or 48th hour. The aberration yields, which account for the number of cells with one, two, or three aberrations, were expressed as percentages of the total number of cells analyzed.

Upon analyzing the induction of the chromosomal aberrations, we observed a chromosomal instability in some cells, expressed as multiple aberrations per cell in all the test systems (Figure 5). In barley and ICR, over 90% of the aberrant cells had only one aberration. Human lymphocytes were more susceptible to the rose essential oil than the other test systems. In barley, after a 4-hour treatment with EsORdm, the total percentages of cells containing more than one aberration ranged from 15.44% to 20.41%. The percentages of cells with two or three aberrations per cell were low, specifically, from 1.02% to 1.34%. Human lymphocytes were more susceptible to EsORdm than the other test systems. The most pronounced chromosomal instability occurred in lymphocytes 4 h after introducing the EsO, where from around 11.10% to 34.40% of the total number of analyzed cells had more than one aberration. The highest genotoxic effect was obtained for a rose essential oil concentration of 500 µg/mL, where three aberrations were detected in 8.30% of the cells (Figure 5C).

The chromosomal instability induced by MNNG was expressed in the induction of 22.79% of the barley cells with two aberrations and 26.70% of the human lymphocytes with two or more aberrations per cell, as compared to 2–6% in ICR mice.

#### 2.2.2. Induction of Micronuclei (MNs)

**Figure 6:**
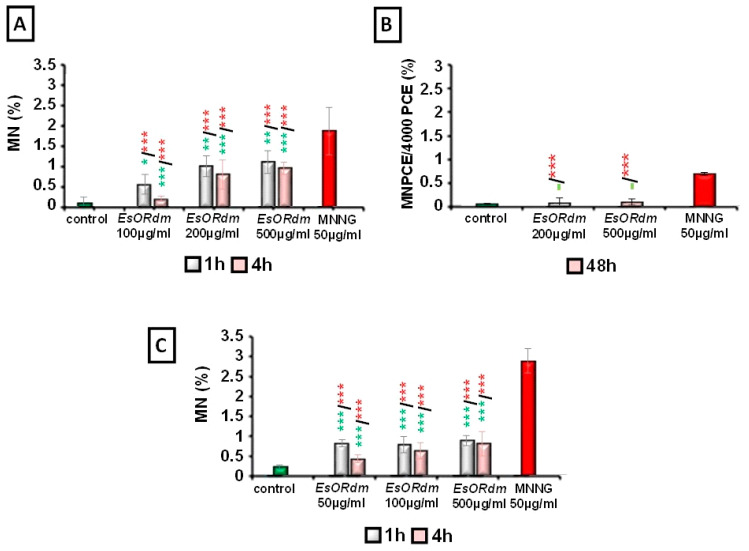
Genotoxic effects of EsORdm assessed based on the values of induced MNs in *H. vulgare* (**A**), mouse bone marrow cells (**B**), and human lymphocytes (**C**); * *p* < 0.05, ** *p* < 0.01, *** *p* < 0.001, and (–) non-significant versus negative control (before the slash) versus positive control, MNNG (after the slash).

A well-expressed statistically significant genotoxic effect was observed both in barley and lymphocyte cells after treatment with all the EsORdm concentrations because the frequency of MNs increased (*p* < 0.05, *p* < 0.01, and *p* < 0.001) compared to the negative control. In the *H. vulgare* test system, the observed MNs, after treatment with EsORdm, ranged from 0.55% ± 0.24 for 100 µg/mL (1 h) to 1.00% ± 0.25 for 200 µg/mL (1 h) and 1.10% ± 0.27 for 500 µg/mL (1 h). After 4 h of treatment, the induction of MNs ranged from 0.20% ± 0.06 (100 µg/mL) to 0.80% ± 0.35 (200 µg/mL) and 0.95% ± 0.15 (500 µg/mL) (Figure 6A).

In human lymphocyte cultures, all three concentrations of EsORdm applied at both treatment durations (1 h and 4 h) showed similar values of MNs. No dose- or duration-dependent relationship was observed (Figure 6C). The MN frequencies, similar to the results for barley, increased significantly (*p* < 0.001), regardless of the treatment period (1 or 4 h), compared to the negative control value. The values were as follows: 0.78% ± 0.08 for 100 µg/mL, 0.74% ± 0.20 for 200 µg/mL, and 0.84% ± 0.13 for 500 µg/mL after 1 h of treatment and 0.40% ± 0.10 for 100 µg/mL, 0.60% ± 0.20 for 200 µg/mL, and 0.78% ± 0.20 for 500 µg/mL after 4 h of treatment.

The MNPCE/PCE frequencies in the bone marrow cells do not show a dose-dependence or a duration-of-treatment effect (*p* > 0.05) between the two EsO concentrations tested (Figure 6B). The percentages of MNs in the peripheral blood PCEs vary from 0.08% ± 0.11 to 0.10% ± 0.06 at 200 and 500 μg/mL of *R. damascena* Mill. essential oil, and these data are statistically indistinguishable from the control value (0.06% ± 0.01; *p* > 0.05) 48 h post IP oil implementation. Therefore, it can be summarized that EsORdm does not cause MN formation, indicating the absence of genotoxicity in vivo.

The genotoxic effects of the EsORdm, as assessed based on MNs, were much lower than those of the MNNG (50 μg/mL) (*p* < 0.001) in all three test systems (1.85% ± 0.57 in *H. vulgare*; 0.69% ± 0.03 in bone marrow cells; 2.80% ± 0.30 in human lymphocytes).

All the concentrations of the *R. damascena* EsO tested showed significant abnormal cytogenetic changes in plant meristems and lymphocyte cells compared to the control groups. This indicates a well-expressed moderate genotoxic effect.

According to the presented data, the genotoxic effect of the EsORdm was the most pronounced in human lymphocytes in vitro.

## 3. Discussion

Research on the safe use of essential oils, especially those widely used in human life, such as rose oils, is essential. The present study provides information on the cytotoxicity and genotoxicity of the essential oil from the Bulgarian *R. damascena* Mill. The cytogenetic study was conducted in three test systems: plant and animal test systems in vivo and human lymphocytes in vitro, as commonly used in genotoxicity studies.

Our analysis showed that the cytotoxic effect of the EsORdm, as assessed based on the MI, was clearly influenced by the sensitivity of the test system, concentrations used in the analyses, and duration of the treatment. EsORdm was not cytotoxic for barley, whereas the ICR mice occupy the intermediate position, where moderate cytotoxicity was reported, depending on the duration of the exposure to the rose essential oil on the bone marrow tissue. Human lymphocytes in vitro were the most sensitive to the cytotoxic action of the *R. damascena* EsO, where the effect clearly depends on the applied concentration. From moderate to high suppression of cellular division, with a dose-dependency, was detected in lymphocyte cells, using the NDI as another marker for cytotoxicity.

The PCE/(PCE + NCE) ratio in the red blood cells of ICR mice is a reliable endpoint that additionally provides valuable information on cytotoxicity. In our *R. damascena* study, this ratio showed a slight but significant reduction in red blood cell proliferation after the application of the rose EsO. That significant decrease in the PCE/(PCE + NCE) ratio in treated mice compared with controls provides evidence of erythropoiesis depression, which led to reduced proliferation of nucleated erythrocyte precursor cells.

These *R. damascena* cytotoxic properties, validated by our results in human lymphocytes and mouse bone marrow cells when the EsO was applied at higher concentrations, are consistent with previous reports showing that essential oil cytotoxicity in mammalian cells is caused by the induction of apoptosis and necrosis [18,35]. *R. damascena* has been reported to have cytotoxic effects on human lung cancer cell lines (A549) and MCF-7 breast cancer cell lines [36]. Zamiri-Akhlaghi et al. [33] found that the *R. damascena* Mill. extract reduced malignant HeLa cell viability in concentration- and time-dependent manners. A recent survey showed that high doses of EsORdm have dose-dependent cytotoxicity toward normal human blood lymphocytes but do not exhibit genotoxic effects [3]. Our results also agree with the studies by Abdel-Hameed et al. [37], who detected that the *R. damascena* Mill. var. *trigintipetala* Dieck EsO demonstrates cytotoxic effects on human peripheral blood lymphocytes (with an IC50 value of higher than 100 μg/mL) and anticancer effects on HepG2 and MCF-7 cell lines. Artun et al. [38] also found that *R. damascena* flower extract had a dose-dependent cytotoxic effect on HeLa and Vero cell lines, with IC50 values of 265 µg/mL and >1000 µg/mL, respectively. Shokrzadeh et al. [4] also reported similar results, with emphasis on antiproliferative activity. The authors reported that EsORdm, at doses from higher than 10 μg/mL to up to 200 μg/mL, had apparent cytotoxic and genotoxic effects on both standard and cancerous cell lines. Additionally, the EsO showed a lower IC50 value in the human cancer NSCLC cell line than in the standard NIH3T3 cell line (non-tumor fibroblast). EsORdm, at a 1% concentration, induced a lethal effect on human lymphocytes after 1 h of incubation, and a similar effect was observed at a 0.1% concentration after 24 h [39]. In this connection, Shokrzadeh et al. [4] suggested using rose EsO as a complementary therapy for cancer, which is also corroborated by Artun et al. [38].

The cytogenetic analysis presented herein showed that the genotoxic effect was well pronounced in both barley and human lymphocytes in vitro (*p* < 0.001) at the concentrations tested, while bone marrow cells did not exhibit this effect. In lymphocytes in vitro, the frequency of CAs increased with increasing applied dose. *R. damascena* EsO also induced multiple aberrations per cell in all the test systems, with the highest level of chromosomal instability observed in lymphocyte cells exposed to the rose essential oil for four hours. It is important to note that the genotoxic effect of the EsORdm is lower than that of the direct mutagen, MNNG.

The CA distribution analysis showed that isochromatid breaks were more prevalent in barley and human lymphocytes. In contrast, centromeres/centromeric fusions and chromatid breaks were more common in the rodent cytogenetic analysis. This result further supports Schubert et al.’s [40] findings that the spectrum of chromatid aberrations varies between plants and mammals.

It is generally accepted that MN induction contributes to chromosomal damage, as Fenech and Morley [41] and Titenko-Holland et al. [42] have noted. The data presented herein revealed substantial increases in the micronuclear frequency in plant meristem cells and lymphocytes in vitro. These results, again, corroborate an earlier study in which Shokrzadeh et al. [4] described the ability of the EsORdm to induce MNs in peripheral blood lymphocytes. The frequency of MNs in their study was observed because of the cytogenetic damage in binucleated lymphocytes when exposed to concentrations of 50–200 μg/mL.

On the other hand, our data are negative for MN formation in mouse PCE cells, providing additional information about the lack of genotoxicity in vivo. This result contrasts with the genotoxic effect observed by us in barley and human lymphocytes in vitro following EsORdm treatment.

The chemical compositions of EsOs are crucial in determining their effects [18], and analyzing them is essential in result interpretation. Ruberto and Baratta [43] and Ipek et al. [44] note that the dominant components in essential oils are responsible for the similarity in their biological activities. This further supports the fact that the strength of their effects is proportional to their concentration, whether tested alone or as a part of an essential oil blend. In our previous study we obtained the chemical composition of the Bulgarian *R. damascena* essential oil [14]. It was detected that the most abundant compounds in the rose essential oil were citronellol and geraniol (overall 44%) and nerol, despite variations in their overall compositions [34]. A study by Berechet et al. [45] on *R. damascena* essential oil reared in Romania reported that only a low percentage of monoterpenes (6.54%, with the main constituents being nerol and p-citronellol) and a large proportion of aliphatic components (85.76%) were identified. *R. damascena* from Morocco [23] contains bioactive compounds, like neral, geranial, phenyl ethyl, kaempferol, and quercetin, with potential antimicrobial properties.

Terpenes in essential oils, on the other hand, can affect cell membrane properties [46], leading to an electrolyte imbalance and cellular death [47]. Geraniol, found in rose absolute oil [3], can increase colon cancer cells’ sensitivity to toxins, while the linalool in the oil has been reported to have anticancer activities together with monoterpenes and sesquiterpenes [48,49]. According to Kim et al. [50], geraniol can inhibit prostate cancer growth by targeting cell cycle and apoptosis pathways. Carnesecchi et al. [51,52] have also previously reported the antiproliferative activity of geraniol in cancer cells, using various assays.

Our cytotoxicity results for EsORdm also align with the findings of Queiroz [53], who demonstrated that geraniol, at concentrations comparable to those described in Gateva et al. [26], exhibited cytotoxicity in human hepatoma cell line HepG2 and human lymphocytes. The susceptibility to geraniol was higher in human lymphocytes than in *H. vulgare* cells [26] and showed a dose-dependent trend.

Jovtchev et al. [31] reported that high doses (500 µg/mL) of geraniol resulted in cytotoxic and genotoxic effects, causing a complete halt in mitotic activity but with no observed induction of chromatid aberrations. Singulani et al. [54] also described that geraniol causes a significant increase in chromosomal damage frequency at an 800 µg/mL dose in V79 cells, which is commensurable, to a certain extent, with our data for 200 and 500 µg/mL doses. A recent study by Mamur [55] also found that geraniol induced genotoxicity in isolated human lymphocytes and MCF-7 cells, especially at higher concentrations (50, 100, and 500 µg/mL), causing increased CA frequency and CA/cell. Still, geraniol did not significantly affect the frequency of the MNi in human lymphocytes. According to the same authors, geraniol also has a cytotoxic effect, reducing the MI by up to 20–100 µg/mL in human lymphocytes and significantly increasing the intensity and moment of the comet tail at 50, 100, and 500 µg/mL. Furthermore, it significantly reduced cell viability in MCF7 cells exposed to between 4 and 500 mg/mL of geraniol for 48 h.

In contrast to our results, a study by Doppalapudi et al. [56] did not report significant induction of bone marrow MNs in mice treated with 375, 750, and 1500 mg/kg of geraniol at any dose level, either in male or female mice, at the 24 or 48-hour time points. However, their findings [56] are consistent with our data on the MN frequency in EsORdm, where no significant induction of MNPCEs in mouse peripheral blood in vivo was observed, suggesting that cyto- and genotoxic effects of EsORdm in vivo are relatively low. The lower genotoxic effect of the rose essential oil in the in vivo rodent test system, compared with that in human lymphocytes in vitro, is probably because of the metabolic features, such as absorption, distribution, and excretion, of the main rose essential oil components. In contrast, metabolic biotransformation is not observed in in vitro tests. Moreover, in vivo assays can provide further insight into genotoxicity detected through in vitro systems [57].

Consistent with the sources mentioned above, geraniol in EsORdm (15.85%), as described in Dobreva et al. [14], is potentially responsible for reduced mitotic activity in bone marrow cells and human lymphocytes.

Eugenol, isoeugenol, and methyleugenol were found to cause cytotoxicity and genotoxicity in rat and mouse hepatocytes, as measured by lactate dehydrogenase release and unscheduled DNA synthesis, respectively [58], as well as inhibitory activity in the cellular growth of cancerous cell lines [28]. Eugenol was found to be genotoxic, as it induced chromosomal aberrations and endoreduplication in V79 cells [59]. The chromatographic profile of the Bulgarian *R. damascena* EsO [14] showed traces and minimal concentrations of eugenol and its analogs.

The cyto- and genotoxic properties of the *R. damascena* essential oil described herein should probably be attributed to the combined effect of the main chemical compounds in the essential oil.

In future research, it would be beneficial to expand the test systems and the concentrations and potentially include the analysis of commercial rose oil products. This would help to enhance our understanding and underscore the importance for conducting comprehensive scientific research.

## 4. Materials and Methods

### 4.1. R. damascena Mill. Essential Oil Extraction

The rose essential oil product from the water–steam distillation of oil-bearing Bulgarian rose *R. damascena* Mill. was provided by the Institute for Roses and Aromatic Plants, in Kazanlak, Bulgaria. The authenticity of the rose species was determined by a specialist from Trakia University, Bulgaria, and the species was preserved in the herbarium of IBER-BAS, with the number SOM 178 483. The rose flowers were collected from the private plantations near Kazanlak. The rose petals were harvested in 2019 and 2020 and distilled using a semi-industrial processing line. Each distillation process involved using 10 kg of raw material for each charge, with a 1:4 hydro-module at a rate of 8–10%, for 150 min. The aromatic water was distilled again using the same equipment, and the EsO of each charge was a mixture of the primary and secondary essential oils in their natural ratio. Water vapor distillation in a Clevenger-type apparatus was performed immediately after collecting the blossoms. The obtained essential oil was collected in the measurement part of the Florentine flask (in mL) [60]. The essential oil was dried with sodium sulfate, filtered, and stored appropriately.

The chromatographic profile of the Bulgarian rose, *R. damascena* Mill., essential oil was determined by GC-FID/MS analysis on an Agilent 7820A GC system (Agilent, Santa Clara, CA, USA) coupled with a flame ionization detector and a 5977B MS detector. The procedure was previously described by Dobreva et al. [14].

### 4.2. Chemicals

The positive control for the experiment was the standard mutagen N-methyl-N’-nitro-N-nitrosoguanidine (MNNG), at a concentration of 50 μg/mL (CAS-Nr.: 70-25-7), which was obtained from Fluka-AG, Buchs, Switzerland. The RPMI 1640 medium used for lymphocyte cultivation was purchased from Sigma-Aldrich, Steinheim, Germany, while the fetal calf serum was from Sigma-Aldrich, Sao Paulo, Brazil. Phyto-hemagglutinin (PHA) and cytochalasin-B were obtained from Sigma-Aldrich, Jerusalem, Israel. Acridine orange, KCl, and acidum aceticum glaciale were purchased from Sigma-Aldrich Chemie GmbH, Merck, Steinheim, Germany. Colchicine was provided by Merck, Darmstadt, Germany, and a 0.9% NaCl solution and gentamicin were from Sopharmacy, Sofia, Bulgaria.

### 4.3. Experimental Design

Analyses were conducted using three different types of test systems to evaluate the cytotoxic and genotoxic effects of EsORdm—barley seed meristems, ICR-strain albino mice, and human lymphocyte cultures. The descriptions of the experimental preparations and the treatment schemes are outlined below.

#### 4.3.1. Plant Test System In Vivo

As a part of the experiment, we utilized root tip meristem cells of the reconstructed karyotype MK 14/2034 of *Hordeum vulgare*. These cells serve as an excellent model of normal cells.

The experiment began with presoaking the seeds in tap water for one hour (h) then germinating them for 19 h in Petri dishes on moist filter paper at 24 °C. Barley seeds were treated with varying concentrations of EsORdm (100, 200, and 500 µg/mL) for 1 h or 4 h. As a positive control, an experimental group was treated with 50 µg/mL of a standard mutagen, 1-methyl-3-nitro-1-nitrosoguanidine, for one hour.

To evaluate chromosomal aberrations (CAs), the seeds were exposed for two hours to a 0.025% colchicine solution saturated with α-bromonaphthalene, after a recovery period ranging from 18 to 30 h at 3-hour intervals (at 24 °C). The root tips were then fixed in ethanol–glacial acetic acid (3:1) and hydrolyzed in 1 N HCl at 60 °C for 9 min, Feulgen stained, macerated in 4% pectinase in distilled water for 14 min, and squashed on slides.

The first-occurring after-treatment mitoses were examined to determine the percentage of metaphases containing CAs, such as chromatid and isochromatid breaks, chromatid translocations, intercalary deletions, and duplications/deletions. The examination was performed on at least 1500 cells at different time intervals (18, 21, 24, 27, and 30 h after treatment).

For micronuclear (MN) scoring, the colchicine treatment was not applied. The frequency of MNs was determined 30 h after treatment.

#### 4.3.2. Animal Test System In Vivo

Eight-week-old male and female ICR-strain albino mice (20.0 ± 1.5 g of b.w.) were delivered from Slivnitza Animal Breeding House, at the Bulgarian Academy of Sciences, Sofia. The animals were transported to the Institute of Biodiversity and Ecosystem Research’s animal house facility and kept for several days under standard laboratory conditions—temperature 20–22 °C, photoperiod from 7 a.m. to 7 p.m., and free access to standard laboratory animal food and water.

The experiments were performed in accordance with Bulgaria’s Directorate of Health Prevention and Humane Behavior toward Animals. The Bulgarian Food Safety Agency (BFSA) published Certificate Number 125 and Standpoint 45/2015 for the use of animals in experiments for the Stephan Angeloff Institute for a five-year period. The Ethical Committee of the Stephan Angeloff Institute approved the experimental design and protocols of the work, with a decision dated 4 October 2020.

The ICR mice were randomly assigned to four experimental groups (eight male/eight female animals each) and kept in standard cages, isolating the control and treatment groups to avoid cross-contamination. All the tested substances were given as a single treatment (0.01 mL/g of bodyweight) by intraperitoneal (IP) injection. EsORdm was diluted in dimethylsulfoxide (DMSO), and the final concentration of DMSO did not exceed 1%.

The following experimental groups (n = 8; 4♂ and 4♀ each) were defined: Group 1. Animals treated with 200 μg/mL of EsORdm. Group 2. Animals treated with 500 μg/mL of EsORdm. Group 3: MNNG (50 μg/mL). Group 4: The untreated control group, which received only 0.9% NaCl. Throughout the experiment, all the animals were observed twice daily, after the initial IP treatment, for any clinical signs of toxicity.

The cytogenetic assay protocol for chromosomal aberrations was conducted for each experimental group, starting at the 24th (4♂ and 4♀) or 48th (4♂ and 4♀) hour following IP treatment with the respective solution [61]. A mitotic inhibitor, colchicine, was injected at a 0.04 mg/g of b.w. dose, one hour before bone marrow cell isolation. Blood smears were prepared before colchicine treatment to score MNs. The animals were euthanized using diethyl ether anesthesia. Bone marrow cells were flushed from the femur and hypotonized in 0.075 M KCl at 37 °C for 15 min. The cell fixation procedure involved using a solution of cold methanol and glacial acetic acid (3:1). The fixed cells were then resuspended and dripped on precleaned and chilled wet glass slides and air dried. The slides were stained in a 5% Giemsa solution (Sigma Diagnostic, Buchs, Switzerland).

##### In Vivo Mammalian Erythrocyte Micronucleus Test

This study conducted the micronucleus test according to OECD test guideline No. 474 for chemical testing. The PCE micronuclear test on mouse cells has been proposed as a rapid and accurate method to detect genotoxicity caused by clastogenic and aneugenic chemical compounds [62]. Peripheral blood samples were collected from all the dose groups 48 h post the initial treatment. A total of 5 µL of peripheral blood was collected from the tail vein and diluted with 45 µL of Sörensen’s phosphate buffer (pH 6.8), and a drop of this solution was spread on a microscope slide. The slides were air dried and fixed for 10 min using absolute methanol. The smeared preparations were stained with acridine orange (AO) and analyzed under a fluorescence microscope Axio Scope A1—Carl Zeiss (Zeiss, Oberkochen, Germany) at 400× magnification, with an FITC 495 nm excitation filter.

The criteria used for identifying MNs and distinguishing them from artifacts in the cytoplasm were described by Schmid [63]. Only monolayers without overlapping cells were targeted for each slide.

#### 4.3.3. Human Lymphocytes In Vitro

To prepare lymphocyte cultures for the present study, peripheral venous blood was obtained from healthy, nonsmoking, and nondrinking donors (men and woman) who were not taking any medication and were aged between 33 and 40. Each culture contained 0.5 mL of lymphocyte suspension, 3.5 mL of RPMI 1640 medium, heat-inactivated fetal bovine serum (12%), 40 mg/mL of gentamycin, and mitogen–phytohemagglutinin (PHA) (0.1%) and was incubated at 37 °C.

The standard method by Evans [64] was used for the cytogenetic analysis of the chromosomal aberrations. Lymphocytes were exposed to three concentrations of EsORdm—50, 100, and 500 µg/mL for either 1 and/or 4 h, administered at the 18th hour (G1) after PHA stimulation. After the treatment, the lymphocytes were rinsed with fresh medium and cultured at 37 °C. At the 72nd hour of the cultivation, the lymphocyte cultures were processed as follows: 0.02% colchicine was added to each sample to stop the mitosis, the cells were hypotonized in 0.56% KCl, fixed in methanol–acetic acid (3:1, *v*/*v*), and stained in 2% Giemsa. Negative control cells were left untreated. As a positive control, cells were treated with MNNG (50 μg/mL) under the same experimental conditions.

To study the induction of MNs, cytochalasin-B (6 μg/mL) was added to each culture at the 44th hour after PHA stimulation, following the cytokinesis-block micronucleus (CBMN) method by Fenech [65]. After 24 h, the lymphocyte cultures were centrifuged, hypotonized with 0.56% KCl, and fixed in a mixture of methanol and acetic acid (3:1, *v*/*v*). The suspension was then dropped onto clean slides and stained with 2% Giemsa.

All the procedures were conducted according to the Declaration of Helsinki (revised in 2013) and approved by the Commission on Ethics and Academic Unity of the Institute of Biodiversity and Ecosystem Research (No. 1, dated 18 February 2022). All the donors provided written informed consent.

### 4.4. Cytogenetic Endpoints

#### 4.4.1. Endpoints for Cytotoxicity

- Mitotic index (MI)

The MI value represents the number of metaphases per 1000 observed cells in each experimental variant. As a cytotoxicity endpoint in plant and lymphocyte test systems, the following formula was used: MI = A/1000, where A is the number of metaphases per 1000 observed cells. For animal cells, the MI was determined by counting the number of dividing cells among 1500 cells per animal [66]. To calculate the mitotic index (MI) in barley and human lymphocytes, 3000 cells were examined for each experimental variant. The frequencies of abnormalities and the MIs in the mice were determined for each animal. The mean ± standard deviation for each experimental variant was calculated.

- PCE/(PCE + NCE) and nuclear division index (NDI)

In addition, the cytotoxic effect of the rose EsO was assessed using the PCE/(PCE + NCE) ratio for each treated ICR mouse.

PCEs—polychromatic erythrocytes;

NCEs—normochromatic erythrocytes.

At least 2000 PCEs per slide, or 4000 per animal, were screened for the incidence of micronucleated immature erythrocytes (MNPCEs) from each slide.

The nuclear division index (NDI), a cell proliferation marker, was used as an additional screening strategy for cytotoxicity in human lymphocyte cells. This biomarker is evaluated using the cytokinesis-blocked micronucleus assay. The formula used is (N1 + 2N2 + 3N3 + 4N4)/N, where N1 to N4 represent the number of cells with 1 to 4 nuclei, and N is the total number of scored cells.

#### 4.4.2. Endpoints for Genotoxicity

- Induction of chromosomal aberrations (CAs)

To assess the genotoxic effect of the EsORdm on all three test systems, the percentages of metaphases with chromosomal aberrations (MwA% ± SD) were calculated. In barley and lymphocyte cultures, the individual types of chromosomal aberrations—chromatid breaks (B’), isochromatid breaks (B”), translocations (T), and intercalary deletions (D) were determined. The main types of chromosomal abnormalities, such as breaks, fragments, and centromere fusions (c/c), were separately scored for each animal in all eight experimental groups.

Over 6000 cells were analyzed in the barley and human lymphocyte tests, while 50 well-scattered metaphases were observed from each mouse.

The plant chromosomes of the reconstructed barley karyotype MK14/2034 were analyzed to identify DNA segments with higher susceptibility to the tested hydrosol sample and/or the mutagen, using “aberration hot spots”. To examine how aberrations are induced in different regions, the metaphase chromosomes of *H. vulgare* were divided into 48 segments of nearly the same size. These segments were numbered based on their location in the standard karyotype [67,68].

- Micronuclear induction (MN)

The percentage of micronuclei (MN% ± SD) was calculated for each treatment variant by analyzing 3000–6000 barley and/or human lymphocyte cells.

In the in vivo animal test system, each slide with peripheral blood was screened for the incidence of micronucleated immature erythrocytes (MNPCEs), with at least 2000 polychromatic erythrocytes (PCEs), or 4000 per animal, being analyzed. To determine the proportion of immature erythrocytes among the total erythrocytes (immature + mature), at least 1000 normochromatic erythrocytes (NCEs) per slide, or 2000 NCEs per animal, were counted.

### 4.5. Statistical Analysis

The experiments were conducted in triplicate, and statistical analysis of the different treatment variants was carried out using one-way ANOVA with a two-tailed Fisher’s exact test. Microsoft Excel 2010 was used for this purpose. Statistical differences were determined as follows: *p* > 0.05 (not significant), * *p* < 0.05 (significant), ** *p* < 0.01 (more significant), and *** *p* < 0.001 (extremely significant). The aberration hotspots in the reconstructed barley karyotype were calculated according to the protocols described by Rieger et al. [69] and Jovtchev et al. [67].

## 5. Conclusions

The present study provides valuable information on the cytotoxic and genotoxic effects of the essential oil from the Bulgarian oil-bearing *R. damascena* in three test systems. The essential oil showed different cytotoxicities, with the specific sensitivity of the cells being a crucial factor in the in vitro and in vivo tests. The effects shown depend on both the concentration and time of exposure. Higher cytotoxicity was found in lymphocyte cultures and from moderate to low in ICR mice. No cytotoxicity toward plant root meristem cells was detected.

All the EsORdm concentrations tested showed moderate genotoxicity in plant meristems and lymphocytes in vitro compared to controls. No genotoxic effect was detected in ICR mice in vivo, and this was confirmed by CA and MN assays. It is essential to note that the effect is much lower than that of the mutagen MNNG.

The results obtained from this study showed that human lymphocytes stood out as the most sensitive test system toward EsORdm. This fact should be kept in mind when developing new products in the fields of advanced pharmaceutics, cosmetics, food technology, and other areas of human life.

So, as a valuable aromatic product of plant origin with multiple uses, *R. damascena* Mill. essential oil should be used in an appropriate concentration range tailored to cellular sensitivity.

## Data Availability

All the obtained data of this research are presented in the manuscript.
